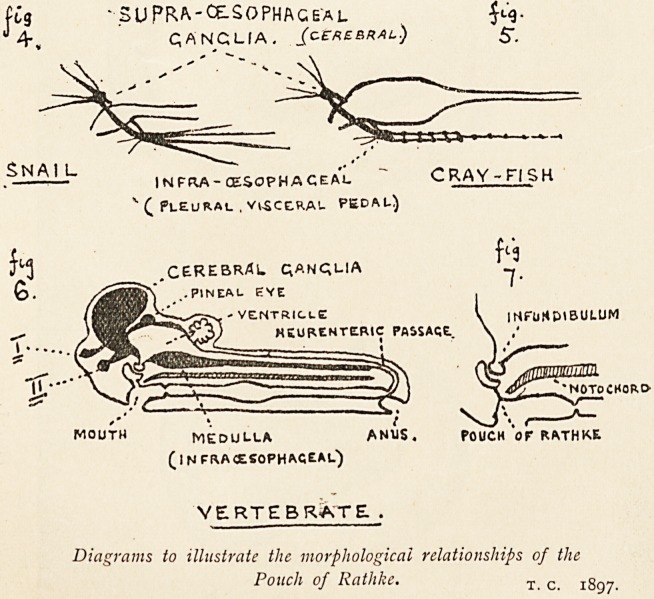# The Morphology and Pathology of the Pharyngeal Pouch of Rathke

**Published:** 1897-12

**Authors:** Thomas Carwardine

**Affiliations:** Assistant-Surgeon, Bristol Royal Infirmary


### THE MORPHOLOGY AND PATHOLOGY OF THE PHARYNGEAL POUCH OF RATHKE.

BY

THOMAS CARWARDINE, M.S. Lond., F.R.C.S. Eng.,
*Assistant-Surgeon, Bristol Royal Infirmary.*

---

THE pharyngeal pouch or bursa pharyngea was first described by Rathke in 1838.[1] It is now a place of great interest both to the surgeon and to the scientist: here post-nasal adenoids have their birthplace, and probably the œsophagus has its ancestry.

The pouch of Rathke is a tubular recess at the upper and posterior wall of the pharynx, not at all infrequent in the adult, commoner in children, and we may say constant in the fœtus. Though veiled as a rule from sight, where there is cleft-palate the pouch can often be seen as a vertical depression, perhaps with well-marked pillars on either side. Fig. 1 shows the pouch in a little boy, aged 3, who was recently under my care. Sometimes the condition is more conspicuous, as in Fig. 2, which is taken from a young woman. It shows a well-marked pharyngeal tonsil of Luschka corresponding to the entrance of the pouch, with deep recesses on either side; and it exemplifies the tendency for the pharyngeal tonsil to be bilobed. Fig. 3 shows the same recess as seen in a child, surrounded by a wreath of adenoids.

The pharyngeal tonsil, sometimes present, contains lymph-follicles and small mucous glands like the faucial tonsils. Moreover, a certain amount of adenoid tissue is normal to the entrance to the pouch of Rathke, as it is to other pouches corresponding to diverticula in the gut of the embryo.

The developmental meaning of this pouch was for long an enigma. In the fœtus, besides the pouch itself, there is a small canal which pierces the sphenoid and leads to the pituitary fossa: it is intersphenoid in position, *i.e.* between the pre- and post-sphenoid developmental centres. According to Meyer,[2] the diverticulum occasionally reaches a large size in the adult, and may be double or septate. Occasionally there is an opening

[1] "Ueber die Entstehung der Glandula pituitaria," *Müller's Archiv.*, 1838.
[2] *Tr. Internat. M. Cong., Lond.*, 1881, iii. 278.

FIG. I. FIG. 2. FIG. 3.

*Cases of Cleft Palate, showing examples of the pouch of Rathke, the pharyngeal tonsil, and adenoids.*

into the sella turcica or into the body of the sphenoid, which one might easily take when small for a nutrient canal.

The pouch is formed from the mouth of the embryo before the communication takes place between the pharynx and fore-gut; and it meets a similar pouch, the infundibulum, from the third ventricle of the brain. In this way the posterior lobe of the pituitary body comes to be formed from the ventricular diverticulum, and the anterior lobe of the pituitary body from the pharyngeal pouch. Moreover, the notochord ceases just posterior to the pouch, where it bends downwards and blends with the buccal epiblast. (See Fig. 7.) The nervous structure of the posterior lobe disappears (although a hollow may persist, lined by ciliated epithelium), whilst the epithelium of the anterior lobe develops into glandular cæcal cavities like those of the thyroid gland; and in some of the larger tubules, *especially those next to the posterior lobe*, cilia may be detected on the cells.[1]

*Diagrams to illustrate the morphological relationships of the Pouch of Rathke.* T. C. 1897.

[1] Quain's *Elements of Anatomy*, 9th Ed., vol. ii. p. 328.

There would thus seem to be a tendency to the union of the mouth with the anterior end of the medullary tube. Such a neurenteric passage has been demonstrated embryologically between the hinder end of the medullary cavity and the hind gut in amphioxus, lizards, etc. Man is reminded of this by the normal presence of the so-called coccygeal gland and by occasional sacro-coccygeal tumours. Through this canalis myeloentericus there is a right of way between the central canal of the spinal cord and the anus. May we not, therefore, concede a right of way also between the pharyngeal and infundibular diverticula which Nature has fenced off for her own private interests? (See Fig. 6.)

In invertebrates the alimentary canal lies for the most part dorsal to the central nervous system, and there is much evidence in favour of the view that the central canal of the nervous system in mammals corresponds to the alimentary canal of invertebrates.

Leydig in 1864 announced his view that the vertebrate brain is the equivalent of the supra- and infra-œsophageal ganglia of invertebrates.[1] In 1882 Prof. Owen suggested that the conario-hypophysial tract (or nervous passage from the pineal gland round the third ventricles to the infundibulum and pituitary body) may have been the means of carrying the anterior part of the alimentary canal from the ventral or hæmal to the dorsal or neural side of the head in an ancestral vertebrate, and that thus the several organs would be brought into corresponding relation with the invertebrate type.[2]

This view has been followed up by Gaskell, who reasonably regards the pouch of Rathke as representing the mouth of the invertebrate, from which the vertebrate was derived.[3] According to Gaskell's view, the epithelial lining of the central nervous system represents the alimentary canal of the crustacean: the œsophagus being represented by the infundibulum, the simple globular non-glandular non-digesting stomach or pouch by the epithelial ventricles of the brain, and the straight narrow intes-

[1] Cited by Gaskell in *Brain*, 1890, xii. 1.

[2] *J. Linnean Soc.* (Zoology), 1883, xvi. 131.

[3] *Brit. M. J.*, 1890, ii. 1341; *Quart. J. Micr. Soc.*, 1890, xxxi. 379, abstract in *J. Roy. Micr. Soc.*, 1890, 701.

tine by the central canal of the cord. The evolution has taken place by the building up of nervous matter around the simple invertebrate stomach. In the lowest vertebrates the lining membrane of the cavities of the brain is more distinct from the nervous matter than in higher vertebrates.

The crura cerebri may be regarded as representing the œsophageal commissures. The supra-œsophageal ganglia, with their volitional, ocular, and olfactory functions, are represented by the cerebral, olfactory, and optic centres; the infra-œsophageal ganglia by the centres of equilibrium in the cerebellum; and the thoracic and ventral ganglia by the vital and locomotor centres in the medulla and cord respectively. I have endeavoured to represent this relationship between the vertebrate spinal and the invertebrate alimentary canals in the accompanying figures 4, 5 and 6, with the aid of sketches made from dissections of the crayfish and snail.

In passing it may be stated that there are the strongest grounds for regarding the pineal gland as a third and unpaired eye.

Experimental evidence bears out this relationship. Removal of the supra-œsophageal ganglion of the crayfish deprives it of will-power. The sub-œsophageal ganglia are the centres for co-ordinating the movements of the limbs. Finally, removal of the otocysts (corresponding to the semi-circular canals) disturbs equilibrium.

Other considerations could be brought to bear upon the question, such as the form of the circle of Willis, and the development of the body of the sphenoid from pre- and post-sphenoid centres, but space is limited.

Pathologically considered, the pouch of Rathke is a little mine of wealth undiscovered. The diseases of the post-nasal region are necessarily obscured by their locality.

*Adenoids.* In 1881 a great discovery was made known when Meyer described the ill-effects of adenoids, and said: "The place of election of the cristate vegetations is the posterior wall of the pharynx, especially its upper curved part."[1] At the

[1] *Loc. cit.*

same meeting the following delightful expression came from Loewenberg: "Le plus grand développement là où se trouve la plus forte agglomération de ce tissu, à savoir la région supérieure et postérieure de l'organe, occupée par la tonsilla pharyngea de Luschka. . . . Je citerai à cet égard . . . où cet organe hypertrophié possédait la configuration et la grosseur d'une forte cerise. . . . En bas, la tumeur était un peu étranglée par une rainure verticale. Il existait, dans ce point, une ouverture dans laquelle les instruments pénétraient profondement; c'était évidemment l'entrée de la bursa pharyngea de Mayer."[1]

*Chronic Catarrh.* The pouch of Rathke is liable to chronic catarrh of a troublesome intractable character, which on the Continent is associated with the name of Tornwaldt.

*Cysts.* A cyst of the bursa pharyngea was first opened by Czermak in 1860, since which time certainly over a hundred cases have been recorded.[2] The cysts may reach the size of a ripe cherry, and are sometimes bilobed like the tonsil itself. They usually contain a colloid or stinking mucoid fluid, and occur towards adult life as a sequel to adenoid hypertrophy. They may be lined by columnar, ciliated or stratified epithelium, or be simple cysts in adenoid tissue. Raulin describes the case of an amateur singer who lost all his tenor notes owing to such a cyst, eight days after the removal of which his tenor notes came back again.[3]

We may now make a provisional classification of cysts of the pharyngeal bursa, thus: (1) Dermoids, or congenital cysts, perhaps containing ciliated epithelium—rare; (2) Cysts of occlusion by adenoid tissue; (3) Cysts of glandular recesses in the pouch; (4) Cystic changes in the lymphoid follicles. Myxomatous cysts in this region are often associated with mucous polypi of the nose.

With regard to dermoids, Mr. Bowlby described such a tumour which appeared to have originated at the junction of the pouch of Rathke with the infundibulum where they form the pituitary body.[4] One part was vascular and fibrous with

1 *Tr. Internat. M. Cong., Lond.*, 1881, iii. 286-7.
2 Cited by Wright in *Med. News*, 1889, lv. 255.
3 *Rev. de Laryngol.* [etc.], 1891, xi. 513.
4 *Tr. Path. Soc. Lond.*, 1885, xxxvi. 35.

cystic epithelial ingrowths, the other part was of bone. Hale White met with a so-called myo-neuroma of the pituitary body, with connective tissue, fat-cells, vessels, nerve-fibres, ganglion cells, and striped muscle.[1] Mr. P. S. Abraham described a hairy dermoid patch as arising from the pouch of Rathke, but after posterior rhinoscopy he altered his views as to the origin of the growth.[2]

*Adenomata.* These resemble the structure of the thyroid gland—"pituitary goitres."[3]

*Fibromata.* It seems very probable that the large pharyngeal or naso-pharyngeal fibrous polypi often arise in association with the pouch of Rathke. This would be sufficient reason for always examining the bases of these polypi microscopically for any sign of a central canal. Cheatham[4] described a pure polypus springing from the pharyngeal tonsil, and also a fibroma springing from the basilar process of the occipital. Gould[5] had a case of fibroma springing from the upper posterior wall of the pharynx and *extending into the sphenoidal sinuses.*[6]

*Fibro-sarcomata.* The same argument applies here as to the foregoing. Fleury[7] described the following: "Polype fongueux (cancéreux), s'étendant à l'arrière-gorge, à la narine droite pénétrant dans la cavité orbitaire, dans le sinus sphénoïdal, et perforant la base du crâne après avoir détruit le corps du sphénoïde."[6]

*Carcinoma.* Dr. Goodhart showed a specimen of carcinoma of the pituitary fossa in a baboon—"probably of the pituitary body." The animal was paralysed before death, and evidenced severe headache by placing the hand to the head and moaning.[8]

*Naso-pharyngeal Tumours and Polypi.* These must be referred to the above-mentioned groups for their pathological positions, and need not be considered under an anatomical heading.

[1] *Tr. Path. Soc. Lond.*, 1885, xxxvi. 37.

[2] *J. Anat. & Physiol.*, 1881, xv. 244.

[3] *Tumours: Innocent and Malignant*, by J. Bland Sutton, 1893, p. 316.

[4] *N. York M. J.*, 1891, liv. 173.

[5] *Lancet*, 1896, ii. 1153.

[6] Evidence perhaps of probable intersphenoid origin.

[7] *Gaz. d. Hôp.*, 1863, xxxvi. 522.

[8] *Tr. Path. Soc. Lond.*, 1885, xxxvi. 36.

I have thus sought to give some uncommon interest to such common affections as adenoids related to the pouch of Rathke. Adenoids, like Cæsar, have been regarded as bungs out of place:

> "Imperious Cæsar, dead and turn'd to clay,
> Might stop a hole to keep the wind away."

But, rightly considered, these obstructions bear the impress of a former glory, when as with a coronet they encircled the archaic mouth.

## Figures and Tables

**FIG. 1. FIG. 2. FIG. 3. f1:**
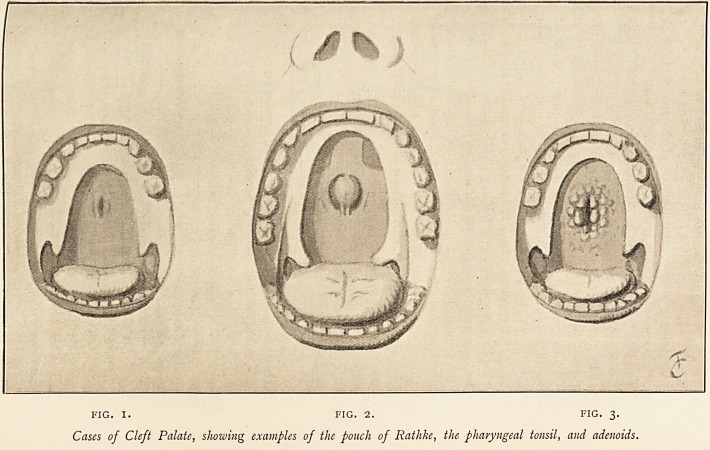


**fig 4. fig. 5. fig 6. fig 7. f2:**